# Combining medial clavicular epiphysis ossification and clavicle length on conventional radiography for forensic age estimation: A multivariable regression approach

**DOI:** 10.1111/1556-4029.70295

**Published:** 2026-03-25

**Authors:** Giorgio De Donno, Federica Mele, Valeria Santoro, Antonio Amato Stabile Ianora, Arnaldo Scardapane, Antonio De Donno

**Affiliations:** ^1^ Interdisciplinary Department of Medicine, Section of Legal Medicine University of Bari Medical School “Aldo Moro” Bari Italy; ^2^ Interdisciplinary Department of Medicine, Section of Diagnostic Imaging University of Bari Medical School “Aldo Moro” Bari Italy; ^3^ Sperimental Medicine Department University of Salento Lecce Italy

**Keywords:** adolescents, adults, age estimation, clavicle length, conventional radiography, forensic radiology, medial clavicular epiphysis, regression analysis

## Abstract

Accurate age estimation is crucial in forensic contexts, yet current methods have limitations. This study evaluates whether integrating clavicle length with medial clavicular epiphysis (MCE) ossification improves age estimation on conventional chest radiographs. A retrospective analysis was performed on 743 chest radiographs from Italian individuals aged 10–30 years. MCE ossification was staged according to Schmeling's classification, and clavicle length was measured. Three multivariable linear regression models were developed, and post‐hoc subgroup analyses were conducted to investigate age‐dependent performance. Results showed that while clavicle length correlated significantly with age in the overall sample (*p* < 0.001), its predictive value was limited to early adolescence (10–15 years) and became non‐significant in the critical forensic window of 16–20 years (*p* = 0.342). Nevertheless, the combined regression model (MCE stage + clavicle length + sex) achieved the best overall performance (*R* ^2^ = 0.685, RMSE = 3.20 years, MAE = 2.51 years; AUC = 0.94, OA = 87.2%). In classifying individuals around the 18‐year threshold, this model significantly outperformed traditional binary cut‐offs and single‐variable models (*p* < 0.001). While the “Stage 4” rule yielded high specificity (98.1%) but low sensitivity (62.5%), the combined regression model achieved a sensitivity of 91.4% with acceptable specificity (80.1%). These findings suggest that transitioning from rigid cut‐offs to statistical modeling provides a more balanced risk assessment, reducing false negatives, although the specific added value of clavicle length diminishes after the pubertal growth spurt.

**Highlights**

- A multivariable model combining MCE stage and clavicle length was evaluated on chest radiographs.
- Clavicle length predicts age in adolescents but adds negligible value at the 18‐year threshold.
- Statistical modeling achieves >90% sensitivity, outperforming the “Stage 4” cut‐off (62.5%).
- Regression reduces false negatives, offering a balanced profile for retrospective analysis.

## 1 INTRODUCTION

Forensic age estimation is a procedure with significant legal and social implications, applicable to both living and deceased individuals [1]. It is particularly essential in contexts such as the identification of skeletal remains [2] and the age assessment of living individuals in migration settings involving unaccompanied minors [1].

Traditional methods based on hand‐wrist radiography [3] or dental development [4] are fundamental but often unsuitable for determining whether an individual has reached the legal age of 18 years. Skeletal maturation of the hand can complete before 18, reducing forensic reliability near this threshold [5]. Likewise, the completion of third molar mineralization alone cannot reliably indicate an age above 18 years, due to its significant variability in developmental timing [6].

Consequently, the evaluation of the medial clavicular epiphysis (MCE) has become a cornerstone of forensic age diagnostics near adulthood. The Study Group on Forensic Age Diagnostics (AGFAD) – an expert consortium providing international recommendations for forensic protocols – emphasizes MCE assessment as an essential element in determining legal majority [7]. The MCE typically fuses between 16 and 21 years, reaching full fusion by 30 years [8]. Achieving Stage 4 ossification, as defined by Schmeling et al. [9], is a cornerstone of current forensic protocols and is widely accepted as a key indicator for suggesting an individual has attained the age of majority [7, 10, 11]. However, despite its high specificity, Stage 4 threshold demonstrates low sensitivity, as many individuals reach this stage well after 18 years.

Computed tomography (CT) is considered the preferred imaging modality for MCE evaluation due to its ability to overcome superimposition [12, 13], but its broader use is limited by cost, radiation dose, and accessibility. Conventional chest radiographs (CR), by contrast, are low‐cost, low‐dose, and often readily available in clinical archives, in accordance with the As Low As Reasonably Achievable (ALARA) principle.

Previous studies have also reported correlations between clavicle length and chronological age [14, 15]. However, since clavicle length strongly correlates with body height [16], it cannot serve as a standalone age indicator. Nevertheless, its potential role as a synergistic variable in combination with MCE ossification has remained underexplored.

The principle of combining multiple sources of developmental information to improve age estimation accuracy is well‐established in the forensic literature. Recent multi‐factorial approaches combining various dental and skeletal indicators have consistently shown superior performance compared to single‐indicator methods [17, 18, 19]. Our study builds upon this principle by investigating, for the first time, the specific synergistic value of clavicle length when combined with MCE staging on conventional radiographs.

## 2 MATERIALS AND METHODS

### 2.1 Study design and population

This retrospective study assessed the combined use of medial clavicular epiphysis (MCE) ossification stage and clavicle length for chronological age estimation on chest radiographs. A series of 743 posteroanterior (PA) chest radiographs obtained from an Italian population aged 10–30 years for clinical purposes was retrospectively evaluated. The study material, collected between 2022 and 2023, was sourced from the archives of three large Italian hospitals: Policlinico di Bari, Ospedale Giovanni XXIII (Bari), and Ospedale di Lecce. The radiographs were originally acquired primarily for evaluating thoracic trauma.

Exclusion criteria were as follows:

- Presence of neoplastic changes or clavicular fractures without the possibility of evaluating the contralateral clavicle.
- Significant overlap of mediastinal structures precludes reliable assessment of the ossification stage.
- Any visible congenital skeletal anomaly

Finally, a total of 666 radiographs were selected, including 449 from males and 217 from females. The sex (male and female), exact date of birth, and date of radiographic examination were recorded, but this information was initially concealed from the examiner.

### 2.2 Radiographic analysis

Two independent forensic radiologists (A.A.S.I., 10 years of experience; A.S., 5 years) analyzed the radiographs using RadiAnt DICOM Viewer (v2024.1 BETA). Each observer independently assigned the MCE ossification stage and measured clavicle length. Discrepancies were solved by consensus before further analysis.

The Schmeling classification [9] was used to determine MCE ossification stage, as this method is recommended by international forensic guidelines [7] and has been widely validated for age estimation on both conventional radiography and CT [11, 20, 21]:

1. Ossification center not yet visible.
2. Ossification center visible but without epiphyseal cartilage ossification.
3. Partial ossification of the epiphyseal cartilage.
4. Complete ossification with visible epiphyseal scar.
5. Complete fusion without visible scar.

If stages differed between sides, the more advanced stage was used.

Clavicle length, measured in centimeters (cm) and recorded to the nearest 0.01 cm, was determined using the ruler tool function in the image analysis software. Clavicle length was defined as the linear distance between the most lateral point of the clavicle, at its articulation with the acromioclavicular (AC) joint, and the most medial point, at its articulation with the sternoclavicular (SC) joint [14]. Measurements were performed on the right clavicle.

### 2.3 Statistical analysis

Analyses were conducted using *Jamovi v2.7.13* and *Microsoft Excel*. Inter‐rater reliability for ordinal MCE staging was assessed using a weighted Cohen's kappa (*κ*), and reliability for continuous clavicle length was assessed using the intraclass correlation coefficient (ICC). Differences in age distribution and clavicle length between sexes were assessed using the Mann–Whitney *U* test and Welch's *t*‐test, respectively. Statistical significance was set at *p* < 0.05.

Three multivariable linear regression models were developed, with chronological age (years) as the dependent variable:

- Model 1: MCE stage + sex
- Model 2: Clavicle length + sex
- Model 3: MCE stage + clavicle length + sex

Sex was coded as male = 0, female = 1. Model fit was evaluated using the coefficient of determination (*R* ^2^), Root Mean Square Error (RMSE), and Mean Absolute Error (MAE). To quantify individual‐level uncertainty, the mean width of the 95% prediction interval (PIW) was calculated for each model.

To investigate the age‐dependent contribution of clavicle length, post‐hoc subgroup analyses were performed using the combined multivariable model (Model 3) on two specific cohorts: 10–15 years (*N* = 161), representing active growth and 16–20 years (*N* = 199), representing the critical forensic window. (See Table S1 for the detailed age distribution of the study sample).

To evaluate diagnostic performance around the 18‐year threshold, classification metrics were computed using predicted ages, defining “adult” = 1 (≥18 years) and “minor” = 0 (<18 years).

Receiver Operating Characteristic (ROC) analysis was performed to evaluate the discriminative ability of each model. AUC values were compared using DeLong's test. Optimal sex‐specific cut‐offs for clavicle length were derived using the Youden Index (*J*). Additionally, the discrimination slope (DS) was calculated as the difference in the mean predicted probabilities between the adult and minor groups derived from logistic regression models.

## 3 RESULTS

### 3.1 Inter‐rater reliability

The agreement between observers was excellent. Weighted Cohen's kappa (*κ*) for MCE ossification staging was 0.92, indicating strong inter‐rater reliability. The Intraclass Correlation Coefficient (ICC) for clavicle length measurements was 0.963, confirming excellent measurement consistency.

### 3.2 Descriptive statistics

Table 1 shows the distribution of chronological age by MCE ossification stage and sex. The mean age approached the 18‐year threshold starting at Stage 3 (17.8 years) but consistently exceeded it only from Stage 4. The Mann–Whitney *U* test indicated no significant difference in stage distributions between males and females (*p* = 0.513).

**TABLE 1:** Distribution of chronological age by MCE ossification stage and sex.

MCE stage	Males (mean ± SD, years)	Females (mean ± SD, years)	Total (mean ± SD, years)
1	13.8 ± 1.4	13.2 ± 1.5	13.6 ± 1.4
2	15.8 ± 1.6	15.3 ± 1.7	15.6 ± 1.6
3	17.9 ± 1.8	17.6 ± 1.9	17.8 ± 1.8
4	20.8 ± 2.1	20.5 ± 2.3	20.7 ± 2.2
5	24.6 ± 3.2	24.3 ± 3.1	24.5 ± 3.2

*Note*: The mean age approached the 18‐year threshold at Stage 3 (17.9 years for males, 17.6 years for females) but consistently exceeded it only starting from Stage 4.

Abbreviation: MCE, medial clavicular epiphysis.

Table 2 summarizes the frequency distribution of MCE stages. Stage 3 was the most frequent category (26.3%), while Stage 5 was the least common (13.8%).

**TABLE 2:** Frequency distribution of MCE ossification stages.

MCE stage	Males (*n*, %)	Females (*n*, %)	Total (*n*, %)
1	70 (15.6%)	25 (11.5%)	95 (14.3%)
2	95 (21.1%)	42 (19.4%)	137 (20.6%)
3	115 (25.6%)	60 (27.6%)	175 (26.3%)
4	100 (22.3%)	67 (30.9%)	167 (25.1%)
5	69 (15.4%)	23 (10.6%)	92 (13.8%)

*Note*: Stage 3 was the most frequent overall (26.3%), while Stage 5 was the least common (13.8%).

Table 3 reports clavicle length values by chronological age and sex. Males exhibited consistently greater clavicle lengths than females (Welch's *t*‐test and Mann–Whitney *U* test, both *p* < 0.001). The difference remained stable after age 16, when mean length plateaued around 17 cm for males and 15.5 cm for females.

**TABLE 3:** Mean clavicle length (cm) by chronological age and sex.

Age (years)	Males (mean ± SD)	Females (mean ± SD)	Total (mean ± SD)
10–12	14.1 ± 1.0	13.0 ± 1.1	13.7 ± 1.1
13–15	15.6 ± 0.9	14.3 ± 1.0	15.1 ± 1.0
16–18	16.8 ± 0.8	15.4 ± 0.9	16.3 ± 0.9
19–21	17.0 ± 0.7	15.6 ± 0.8	16.6 ± 0.8
22–25	17.1 ± 0.6	15.7 ± 0.7	16.7 ± 0.7
26–30	17.1 ± 0.5	15.6 ± 0.6	16.7 ± 0.6

*Note*: Males exhibited consistently greater clavicle lengths than females (*p* < 0.001). Mean values plateaued after age 16.

**FIGURE 1:**
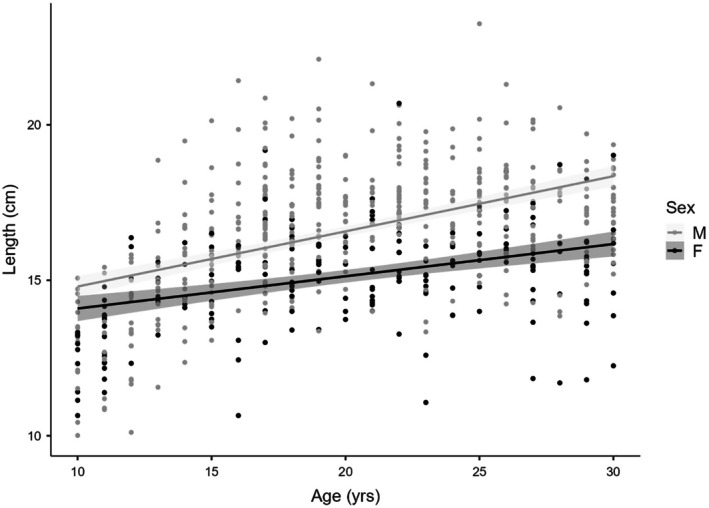
Scatterplot of clavicle length by chronological age, stratified by sex. The dots represent individual measurements. The solid lines indicate the linear regression trends for males (light gray) and females (dark gray), and the shaded areas represent the standard error.

Figure 1 illustrates the distribution of clavicle length by chronological age for males and females. The scatterplot demonstrates the linear growth trends (solid lines), confirming that males consistently exhibit greater clavicle lengths than females.

### 3.3 Regression analysis and prediction uncertainty

**FIGURE 2:**
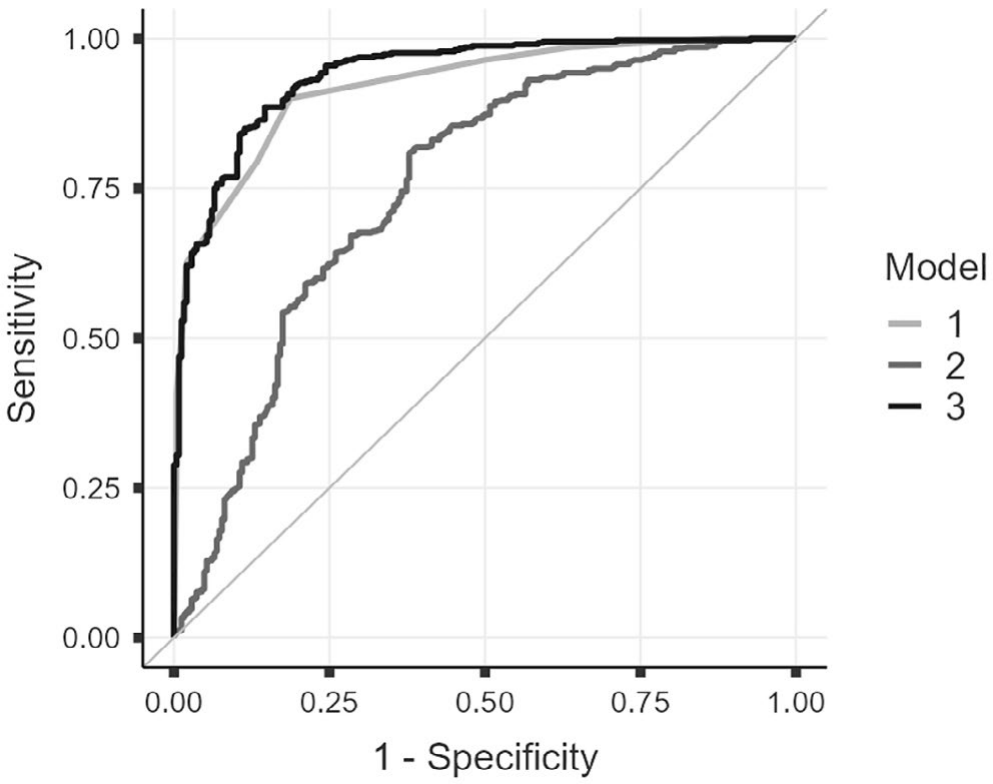
Receiver Operating Characteristic (ROC) curves for Models 1 (MCE stage + sex), 2 (clavicle length + sex), and 3 (combined model). The combined model achieved the highest area under the curve (AUC = 0.94), indicating superior discriminative performance.

Three multivariable linear regression models were tested using chronological age as the dependent variable: Model 1 (MCE stage + sex), Model 2 (Clavicle length + sex), and Model 3 (MCE stage + clavicle length + sex). The regression equations and model performance metrics are summarized in Table 4. All models were statistically significant (*p* < 0.001). Model 3 provided the best fit, with the highest explained variance (*R* ^2^ = 0.685) and the lowest prediction error (RMSE = 3.20 years, MAE = 2.51 years). Consistent with the regression metrics, the uncertainty quantification showed that Model 3 achieved the narrowest mean 95% Prediction Interval Width (PIW) of 12.6 years, compared to 13.2 years for Model 1 (Figure 2).

**TABLE 4:** Regression model performance metrics.

Model	Equation	*R* ^2^	RMSE (years)	MAE (years)	Mean 95% PIW
Model 1 (stage + sex)	Age = 9.66 + 3.31 × stage −0.20 × sex	0.655	3.35	2.60	13.2
Model 2 (length + sex)	Age = −1.37 + 1.29 × length + 1.87 × sex	0.201	5.09	4.25	20.0
Model 3 (combined)	Age = 1.66 + 3.04 × stage +0.53 × length + 0.58 × sex	0.685	3.20	2.51	12.6

*Note*: Model 3 provided the best fit, with the highest explained variance and lowest prediction error.

Abbreviations: 95% PIW, 95% prediction interval width; MAE, mean absolute error; RMSE, root mean square error.

### 3.4 Subgroup analysis

In the 10–15 years subgroup (*N* = 161), clavicle length emerged as a highly significant predictor (*p* < 0.001). Conversely, in the 16–20 years subgroup (*N* = 199), clavicle length was not statistically significant (*p* = 0.342).

### 3.5 Diagnostic performance and classification around the 18‐year threshold

The results of the diagnostic performance comparison are consolidated in Table 5.

**TABLE 5:** Diagnostic performance comparison between simple cut‐off rules and multivariable regression models for discriminating adults (≥18 years).

Approach	Method	Sensitivity (%)	Specificity (%)	Overall accuracy (%)	AUC
Simple cut‐offs	MCE stage **≥**4	62.5	98.1	75.7	0.92
	Clavicle length **≥** threshold^a^	81.4	61.8	74.2	0.75
Regression models	Model 1 (stage + sex)	90.0	81.3	86.8	0.92
	Model 2 (length + sex)	95.0	32.5	71.9	0.75
	Model 3 (combined)	91.4	80.1	87.2	0.94

^a^ Optimal thresholds derived from Youden Index: **≥**16.09 cm for males and **≥**14.49 cm for females.

The traditional forensic rule, “MCE Stage ≥4”, demonstrated the highest specificity (98.1%) but critically low sensitivity (62.5%), resulting in a moderate overall accuracy (75.7%). To ensure a fair comparison, optimized clavicle length cut‐offs were derived using the Youden Index: ≥16.09 cm for males and ≥14.49 cm for females. This optimized threshold provided a sensitivity of 81.4% but a lower specificity of 61.8%, with an overall accuracy of 74.2%.

Multivariable regression models consistently demonstrated superior overall performance. ROC analysis (Figure 2) confirmed that the combined Model 3 achieved the highest discriminative performance (AUC = 0.94), outperforming Model 1 (AUC = 0.92) and Model 2 (AUC = 0.75). When predicted ages were dichotomized at the 18‐year threshold, Model 3 achieved the highest overall accuracy (87.2%), with a well‐balanced profile of sensitivity (91.4%) and specificity (80.1%). This performance outperformed Model 1 (86.8%) and was notably superior to all simple cut‐off approaches. DeLong's test confirmed that the AUC of Model 3 was statistically higher than that of Model 1 (*p* < 0.001), while the overall accuracy (87.2%) surpassed the traditional Stage 4 cut‐off (75.7%). Finally, the discrimination slope (DS) increased from 0.561 for Model 1 to 0.602 for Model 3, indicating a slight improvement in the separation between minors and adults.

## 4 DISCUSSION

The assessment of the medial clavicular epiphysis (MCE) is a cornerstone of forensic age estimation for individuals around the legal age of majority. This is because other widely used skeletal and dental indicators, such as the hand‐wrist and third molars, may have already completed their development before an individual reaches 18 years of age, thus limiting their discriminatory power for this specific legal threshold [3, 4, 5, 6].

### 4.1 Addressing conventional radiography limitations and the role of CT

The assessment of MCE ossification using conventional radiography (CR) is known to face challenges due to superimposition from adjacent structures [22, 23]. In our initial sample, this issue precluded a reliable assessment in 7.81% of cases. To overcome these challenges, cross‐sectional imaging, particularly computed tomography (CT), is now widely regarded as the method of choice for MCE assessment [10, 21], with some authors considering it the “exclusive method of choice” for de novo medico‐legal evaluations [22]. Despite these limitations, conventional radiography of the clavicle retains significant utility in specific forensic scenarios. As demonstrated in our study, a reliable assessment was possible in over 92% of cases. Its primary value lies in the evaluation of pre‐existing radiographic material, such as chest X‐rays obtained for clinical reasons (e.g., tuberculosis screening in unaccompanied minors), where further radiation exposure is not ethically or legally justifiable [22]. In these contexts, CR serves as a crucial, low‐dose, and readily available source of information. Therefore, while CT is the gold standard for prospective forensic age assessments, CR remains an indispensable tool for retrospective analysis, fully aligning with the ALARA principle. Our choice to utilize CR in this study was driven by the rationale of developing a robust model for re‐evaluating existing clinical images, thereby maximizing the diagnostic potential of data that is already available.

### 4.2 The age‐dependent contribution of clavicle length

It is important to acknowledge that clavicle length is primarily correlated with overall body dimensions, particularly height [16], which limits its direct applicability as a sole age indicator. Our results confirmed that, as a standalone predictor (Model 2), it demonstrated significant limitations, including critically low specificity (32.5%), rendering it unsuitable for reliable standalone medico‐legal use. While our general regression analysis (10–30 years) showed a statistical improvement with the inclusion of clavicle length (*p* < 0.001), the post‐hoc subgroup analysis clarified that clavicle length was a highly significant predictor in the 10–15 years age group, reflecting active skeletal growth. Conversely, in the critical forensic window of 16–20 years, clavicle length was not statistically significant (*p* = 0.342). This indicates that the value of clavicle length is strictly age‐dependent: it aids in describing the general developmental trend in early adolescence but provides negligible added value for discriminating adults from minors once the growth plateau is reached. This finding aligns with observations by De Tobel et al. [19] who reported that adding anthropometric variables to ossification stages yields diminishing returns in late adolescence.

### 4.3 Regression models vs. binary cut‐offs

Consistent with a large body of literature based on both conventional radiography [11, 20] and computed tomography [10, 21], our analysis confirmed that MCE Stage 4 is a highly specific indicator for the attainment of legal majority. In numerous studies, the minimum age observed for Stage 4 is consistently at or above 18 years, making it a reliable marker for confirming adulthood. However, its critical limitation is its low sensitivity (approx. 62%), meaning a large proportion of actual adults may not have reached this stage. This finding of low sensitivity is strongly consistent with the broader forensic literature. Numerous studies report a wide age range for individuals in Stage 3, with maximum ages frequently extending well into the late 20s or even 30s [11, 20, 24, 25, 26]. The development of clavicular substaging systems was, in fact, a direct response to this challenge [27]. Our study suggests that multivariable statistical modeling may offer advantages over traditional classification based on developmental cut‐offs. While the “Stage 4” rule demonstrated high specificity, it missed nearly 40% of adults in our sample. In contrast, the regression‐based approach (Model 3) provides quantitative age estimation and achieved a sensitivity of >90% while maintaining acceptable specificity (~80%). These findings indicate that moving from threshold‐based rules toward statistical modeling could provide a more balanced risk assessment in forensic practice, reducing the rate of false negatives.

### 4.4 Comparison with multi‐factorial approaches

Finally, it is important to contextualize our results within the broader landscape of forensic age estimation. The mean width of the 95% prediction interval (PIW) in our study (12.6 years) is relatively wide compared to multi‐factorial approaches. For instance, studies combining dental and hand‐wrist maturity [17, 18], or integrating third molars, wrist, and clavicle using MRI [19], reported prediction intervals of approximately 4–6 years. This discrepancy is attributable to the reliance on a single anatomical site (the clavicle) and the inherent limitations of 2D radiography compared to 3D imaging. Therefore, the method proposed here is not intended to replace multi‐factorial MRI/CT protocols. Instead, it offers a statistically rigorous framework for the retrospective evaluation of existing chest radiographs.

While this study provides valuable insights, some limitations should be acknowledged. First, the study population was limited to Italian individuals, which may limit the generalizability of our findings. Furthermore, information regarding socioeconomic status (e.g., HDI ranking) and specific details on potential underlying medical conditions known to influence growth and development were not available in this retrospective dataset. Second, the data were collected retrospectively from conventional chest radiographs. Finally, despite careful assessment, the inherent limitations of CR due to structural superimposition present a challenge compared to CT, which is widely considered the preferred modality for de novo forensic age assessments.

## 5 CONCLUSIONS

This study assessed the utility of combining the MCE ossification stage and clavicle length for forensic age estimation on conventional radiographs. Subgroup analyses clarified that clavicle length is a strong predictor of age only during active growth (10–15 years) and does not provide a specific diagnostic advantage for discriminating between minors and adults in the critical 16–20 years window. However, the study provides evidence that multivariable statistical modeling offers distinct methodological advantages over traditional binary cut‐offs. The proposed model demonstrated robust statistical performance (*R* ^2^ = 0.685, RMSE = 3.20 years, MAE = 2.51 years; AUC = 0.94, OA = 87.2%), allowing forensic practitioners to significantly increase sensitivity (>90%) and reduce the rate of false negatives compared to the rigid “Stage 4” rule. While conventional radiography yields wider prediction intervals than multi‐factorial 3D imaging, the proposed model offers a statistically rigorous framework for analyzing existing X‐ray evidence. Future studies on diverse populations are warranted to validate the proposed models and confirm their applicability in broader forensic contexts.

## CONFLICT OF INTEREST STATEMENT

The authors declare that they have no competing interests.

## ETHICS APPROVAL STATEMENT

This retrospective study was performed in accordance with the ethical standards of the 1964 Helsinki Declaration and its later amendments. The research protocol was approved by the Ethics Committee of Giovanni Paolo II Hospital, Bari, Italy.

## CONSENT TO PARTICIPATE

The need for individual informed consent was waived by the ethics committee due to the retrospective and fully anonymized nature of the study.

## CONSENT FOR PUBLICATION

Given the retrospective nature of this study, the need for consent for publication was waived by the ethics committee. All data were anonymized to protect the privacy of the participants.

## Supporting information


Table S1

## Data Availability

The datasets used and/or analyzed during the current study are available from the corresponding author on reasonable request.

## References

[1] Ritz‐Timme S , Cattaneo C , Collins MJ , Waite ER , Schütz HW , Kaatsch H‐J , et al. Age estimation: the state of the art in relation to the specific demands of forensic practise. Int J Legal Med. 2000;113:129–136. 10.1007/s004140050283 10876982

[2] Franklin D . Forensic age estimation in human skeletal remains: current concepts and future directions. Legal Med. 2010;12:1–7. 10.1016/j.legalmed.2009.09.001 19853490

[3] Tisè M , Mazzarini L , Fabrizzi G , Ferrante L , Giorgetti R , Tagliabracci A . Applicability of Greulich and Pyle method for age assessment in forensic practice on an Italian sample. Int J Legal Med. 2011;125:411–416. 10.1007/s00414-010-0541-6 21221985

[4] De Donno A , Angrisani C , Mele F , Introna F , Santoro V . Dental age estimation: Demirjian's versus the other methods in different populations. A literature review. Med Sci Law. 2021;61:125–129. 10.1177/0025802420934253 33591866

[5] Chaumoitre K , Saliba‐Serre B , Adalian P , Signoli M , Leonetti G , Panuel M . Forensic use of the Greulich and Pyle atlas: prediction intervals and relevance. Eur Radiol. 2017;27:1032–1043. 10.1007/s00330-016-4466-4 27357132

[6] Olze A , Solheim T , Schulz R , Kupfer M , Schmeling A . Evaluation of the radiographic visibility of the root pulp in the lower third molars for the purpose of forensic age estimation in living individuals. Int J Legal Med. 2010;124:183–186. 10.1007/s00414-009-0415-y 20111870

[7] Schmeling A , Dettmeyer R , Rudolf E , Vieth V , Geserick G . Forensic age estimation: methods, certainty, and the law. Dtsch Arztebl Int. 2016;113(4):44–50. 10.3238/arztebl.2016.0044 26883413 PMC4760148

[8] Scheuer L , Black S . Developmental juvenile osteology. London, UK: Academic Press; 2000.

[9] Schmeling A , Schulz R , Reisinger W , Muhler M , Wernecke K‐D , Geserick G . Studies on the time frame for ossification of the medial clavicular epiphyseal cartilage in conventional radiography. Int J Legal Med. 2004;118:5–8. 10.1007/s00414-003-0404-5 14534796

[10] Hermetet C , Saint‐Martin P , Gambier A , Ribier L , Sautenet B , Rérolle C . Forensic age estimation using computed tomography of the medial clavicular epiphysis: a systematic review. Int J Legal Med. 2018;132:1415–1425. 10.1007/s00414-018-1847-z 29713801

[11] Wittschieber D , Ottow C , Schulz R , Püschel K , Bajanowski T , Ramsthaler F , et al. Forensic age diagnostics using projection radiography of the clavicle: a prospective multi‐center validation study. Int J Legal Med. 2016;130:213–219. 10.1007/s00414-015-1285-0 26518299

[12] Schulz R , Mühler M , Reisinger W , Schmidt S , Schmeling A . Radiographic staging of ossification of the medial clavicular epiphysis. Int J Legal Med. 2008;122:55–58. 10.1007/s00414-007-0210-6 17940787

[13] Wittschieber D , Schulz R , Vieth V , Küppers M , Bajanowski T , Ramsthaler F , et al. The value of sub‐stages and thin slices for the assessment of the medial clavicular epiphysis: a prospective multi‐center CT study. Forensic Sci Med Pathol. 2014;10:163–169. 10.1007/s12024-013-9511-x 24277267

[14] McGraw MA , Mehlman CT , Lindsell CJ , Kirby CL . Postnatal growth of the clavicle: birth to 18 years of age. J Pediatr Orthop. 2009;29:937–943. 10.1097/BPO.0b013e3181c11992 19934713 PMC2806601

[15] Hosseinzadeh P , Pokala N , Meyer Z , Minaie A , Brea C , Gonzalez D , et al. Clavicles continue to grow beyond skeletal maturity: radiographic analysis of clavicle length in adolescents and young adults. J Pediatr Orthop B. 2020;29:195–199. 10.1097/BPB.0000000000000644 31356506

[16] Trangadia M , Gupta B . Estimation of stature from the length of clavicle in adult (post‐fusion age) in Saurashtra region of Gujarat. J Forensic Med Toxicol. 2020;37:14–17. 10.5958/0974-4568.2020.00003.4

[17] Bleka Ø , Rolseth V , Dahlberg PS , Saadé A , Saadé M , Bachs L . BioAlder: a tool for assessing chronological age based on two radiological methods. Int J Legal Med. 2019;133:1177–1189. 10.1007/s00414-018-1959-5 30386872

[18] Bleka Ø , Wisløff T , Dahlberg PS , Rolseth V , Egeland T . Advancing estimation of chronological age by utilizing available evidence based on two radiographical methods. Int J Legal Med. 2019;133:217–229. 10.1007/s00414-018-1848-y 29736772

[19] De Tobel J , Fieuws S , Hillewig E , Phlypo I , van Wijk M , de Haas MB , et al. Multi‐factorial age estimation: a Bayesian approach combining dental and skeletal magnetic resonance imaging. Forensic Sci Int. 2020;306:110054. 10.1016/j.forsciint.2019.110054 31778924

[20] Schulz R , Mühler M , Mutze S , Schmidt S , Reisinger W , Schmeling A . Studies on the time frame for ossification of the medial epiphysis of the clavicle as revealed by CT scans. Int J Legal Med. 2005;119:142–145. 10.1007/s00414-005-0529-9 15711799

[21] Ufuk F , Agladioglu K , Karabulut N . CT evaluation of medial clavicular epiphysis as a method of bone age determination in adolescents and young adults. Diagn Interv Radiol. 2016;22:241–246. 10.5152/dir.2016.15355 27015321 PMC4859740

[22] Wittschieber D , Ottow C , Vieth V , Küppers M , Schulz R , Hassu J , et al. Projection radiography of the clavicle: still recommendable for forensic age diagnostics in living individuals? Int J Legal Med. 2015;129:187–193. 10.1007/s00414-014-1067-0 25135751

[23] Shedge R , Kanchan T , Warrier V , Dixit SG , Krishan K . Forensic age estimation using conventional radiography of the medial clavicular epiphysis: a systematic review. Med Sci Law. 2021;61:138–146. 10.1177/0025802420988223 33541216

[24] Marera DO , Satyapal KS . Fusion of the medial clavicular epiphysis in the south African and Kenyan populations. Int J Morphol. 2018;36:1101–1107. 10.4067/S0717-95022018000301101

[25] Brown AA , Derkyi‐Kwarteng L , Amonoo‐Kuofi HS . Study on the time frame for ossification of the medial clavicular epiphyseal cartilage by X‐ray in Ghanaian students. Int J Morphol. 2013;31:491–496. 10.4067/S0717-95022013000200021

[26] Garamendi PM , Landa MI , Botella MC , Alemán I . Forensic age estimation on digital X‐ray images: medial epiphyses of the clavicle and first rib ossification in relation to chronological age. J Forensic Sci. 2011;56(1):3–12. 10.1111/j.1556-4029.2010.01626.x 21155800

[27] Kellinghaus M , Schulz R , Vieth V , Schmidt S , Schmeling A . Forensic age estimation in living subjects based on the ossification status of the medial clavicular epiphysis as revealed by thin‐slice multidetector computed tomography. Int J Legal Med. 2010;124:149–154. 10.1007/s00414-009-0398-8 20013127

