# Supplementary material for: Combining medial clavicular epiphysis ossification and clavicle length on conventional radiography for forensic age estimation: A multivariable regression approach
Source: J Forensic Sci. 2026 Mar 25;71(3):1091–8. doi: 10.1111/1556-4029.70295 (PMC13139803; doi:10.1111/1556-4029.70295)
Supplement: Supplementary file 1 — Table S1 [file JFO-71-1091-s001.docx]

TABLE S1 Detailed frequency distribution of the study sample by chronological age and sex. The specific subgroups used for the post-hoc analysis (10–15 years and 16–20 years) are highlighted.

| **Age (years)** | **Males (n)** | **Females (n)** | **Total (n)** |
| --- | --- | --- | --- |
| 10 | 14 | 9 | 23 |
| 11 | 13 | 16 | 29 |
| 12 | 18 | 4 | 22 |
| 13 | 21 | 9 | 30 |
| 14 | 18 | 6 | 24 |
| 15 | 23 | 10 | 33 |
| **Subtotal (10–15 years)** | **107** | **54** | **161** |
|  |  |  |  |
| 16 | 17 | 11 | 28 |
| 17 | 45 | 12 | 57 |
| 18 | 31 | 17 | 48 |
| 19 | 32 | 9 | 41 |
| 20 | 19 | 6 | 25 |
| **Subtotal (16–20 years)** | **144** | **55** | **199** |
|  |  |  |  |
| 21 | 16 | 16 | 32 |
| 22 | 25 | 14 | 39 |
| 23 | 21 | 10 | 31 |
| 24 | 17 | 11 | 28 |
| 25 | 22 | 9 | 31 |
| 26 | 26 | 8 | 34 |
| 27 | 19 | 13 | 32 |
| 28 | 12 | 5 | 17 |
| 29 | 17 | 13 | 30 |
| 30 | 23 | 9 | 32 |
| TOTAL | 449 | 217 | 666 |
